# Hemophagocytic syndrome caused by Epstein–Barr virus and cytomegalovirus infection during neoadjuvant chemoradiotherapy for rectal cancer: a case report

**DOI:** 10.3389/fmed.2026.1722216

**Published:** 2026-03-03

**Authors:** Kou Kanesada, Kazuhiko Yoshimatsu, Shuya Yano, Masaharu Higashida, Toshimasa Okada, Shunji Endo, Yoshinori Fujiwara, Tomio Ueno

**Affiliations:** Department of Digestive Surgery, Kawasaki Medical School, Kurashiki, Japan

**Keywords:** cytomegalovirus infection, Epstein–Barr virus, hemophagocytic syndrome, neoadjuvant chemoradiotherapy, rectal cancer

## Abstract

**Background:**

Immune dysregulation and excessive inflammatory responses can lead to hemophagocytic syndrome (HPS) involving autologous blood cell phagocytosis, with fatal outcomes occurring in some cases. This case report describes an 80-year-old man who was simultaneously diagnosed with diffuse large B-cell lymphoma (DLBCL) and rectal cancer and developed HPS during neoadjuvant chemotherapy for the latter.

**Case description:**

Treatment for DLBCL was initiated first, and six courses of rituximab, cyclophosphamide, doxorubicin, vincristine, and prednisone (R-CHOP) therapy were administered, which led to a clinical complete response of the lymphoma lesions. Following the completion of DLBCL treatment, preoperative chemoradiotherapy with tegafur–uracil/leucovorin (UFT/UZEL) was initiated for rectal cancer. On Day 18, a fever of 38.3 °C developed. Blood tests conducted on Day 24 revealed Grade 4 neutropenia and Grade 4 thrombocytopenia. Granulocyte colony-stimulating factor (G-CSF) preparation, antibiotic therapy, and recombinant human soluble thrombomodulin (rTM) were initiated as disseminated intravascular coagulopathy (DIC) therapy. A poor therapeutic response was achieved, and acute respiratory distress syndrome (ARDS) developed on Day 34. Imaging of the biopsied bone marrow confirmed that hemophagocytosis by macrophages was occurring. The patient was ultimately diagnosed with HPS. Epstein–Barr virus (EBV) and cytomegalovirus (CMV) infections were identified, and treatment to combat the infections was initiated; however, the patient passed away on Day 37.

**Conclusion:**

It is important to consider the possibility of HPS, and diagnosis and treatment initiation should occur in a timely manner when fever of an unknown origin and decreased blood cell counts are observed during malignant disease treatment.

## 1 Introduction

Hemophagocytic syndrome (HPS) is a disease characterized by the hemophagocytosis of autologous blood cells by histiocytes and macrophages in the reticular system, including the bone marrow and lymph nodes. HPS can manifest as a variety of symptoms, including fever, lymphadenopathy, hepatosplenomegaly, pancytopenia, abnormal coagulation, liver dysfunction, elevated concentrations of lactate dehydrogenase, hypertriglyceridemia, and hyperferritinemia (1). HPS often occurs as a result of immune dysregulation and an excessive inflammatory response, and the outcomes can be fatal in some cases (2). However, owing to the fact that the symptoms of HPS are nonspecific, achieving a definitive diagnosis can be challenging. This case report describes a patient with HPS who experienced a poor outcome during preoperative chemoradiotherapy (CRT) for rectal cancer, which resulted in marked hematopenia and multiorgan failure induced by infection with Epstein–Barr virus (EBV) and cytomegalovirus (CMV).

## 2 Case description

**Figure 1:**
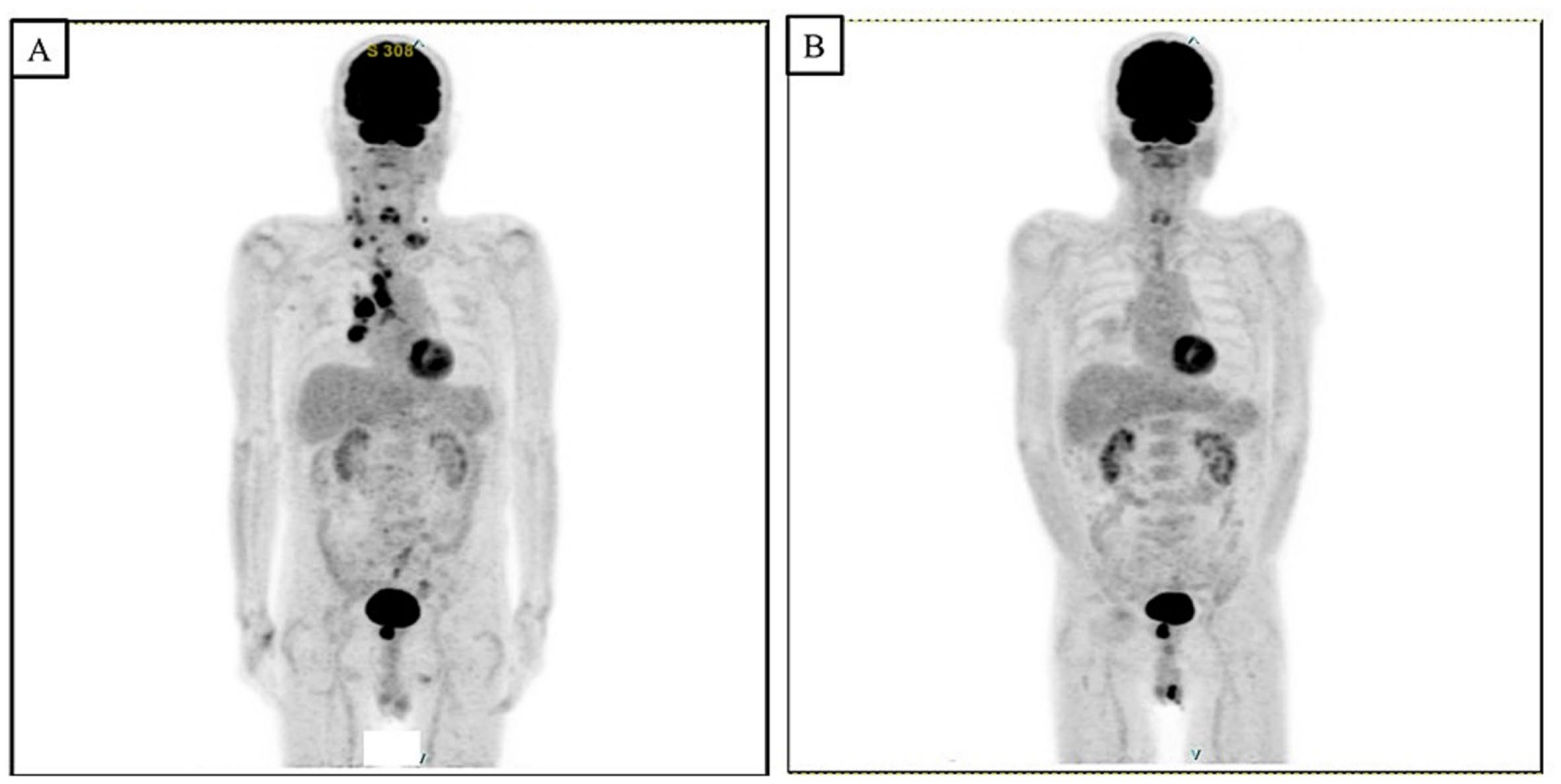
PET-CT scan revealed multiple FDG accumulations mainly in the bilateral neck, supraclavicular fossa, mediastinum, and right hilar lymph nodes **(A)**. After treatment for DLCBL with a total of six courses of R-CHOP, PET-CT scan showed that accumulation of the FDG in the lymphoma lesions had disappeared, and the treatment effect was a clinical complete response **(B)**.

An 80-year-old man presented with anorexia and bloody stools. His medical history included cerebral infarction and type 2 diabetes, and there was no notable family history. The colonoscopy revealed an irregularly ulcerative tumor in the lower rectum. A biopsy revealed a well-differentiated carcinoma. Computed tomography (CT) revealed the presence of enlarged lymph nodes outside the region affected by rectal cancer, and positron emission tomography–CT (PET-CT) revealed multiple sites of fluorine-18-fluodeoxyglucose (FDG) accumulation, predominantly in the bilateral neck, supraclavicular fossa, mediastinum, and right hilar lymph nodes (Figure 1A). Blood tests showed an elevated concentration of soluble interleukin 2 receptor (1,255 U/mL). The right cervical lymph node was biopsied, and the histopathological examination led to the diagnosis of diffuse large B-cell lymphoma (DLBCL). The clinical stage of the rectal cancer was determined to be T3N0M0, Stage 2 (based on the criteria of the Japanese Society for Cancer of the Colon and Rectum, 9th edition), and the malignant lymphoma was diagnosed as cluster of differentiation 5-positive (CD5^+^) DLBCL (germinal center B-cell (GCB) type; Lugano classification: Stage 3). Treatment for DLBCL was initiated first, and six courses of rituximab, cyclophosphamide, doxorubicin, vincristine, and prednisone (R-CHOP) therapy were administered (rituximab: 570 mg/body, cyclophosphamide: 1,130 mg/body, doxorubicin: 75 mg/body, vincristine: 2 mg/body, and prednisolone: 60 mg/body). PET-CT conducted post-treatment revealed that FDG accumulation in the lymphoma lesions had disappeared (Figure 1B), and a clinical complete response was achieved. Following the completion of treatment for DLBCL, preoperative CRT was initiated for rectal cancer, with the chemoradiotherapeutic regimen comprising a combination of tegafur–uracil (UFT; 400 mg/day)/leucovorin (UZEL; 75 mg/day) and radiation therapy (45 Gy).

## 3 Diagnostic assessment

**Figure 2:**
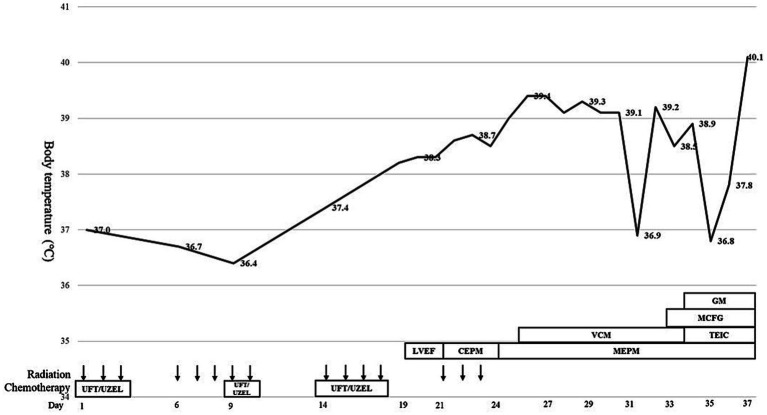
This chart shows the course of treatment after the start of chemoradiotherapy for rectal cancer. Body temperature indicates the highest temperature during the day. LVEF, levofloxacin; CEPM, cefepime; MEPM, meropenem; VCM, vancomycin; TEIC, teicoplanin; MCFG, micafungin; GM, gentamicin.

**Figure 3:**
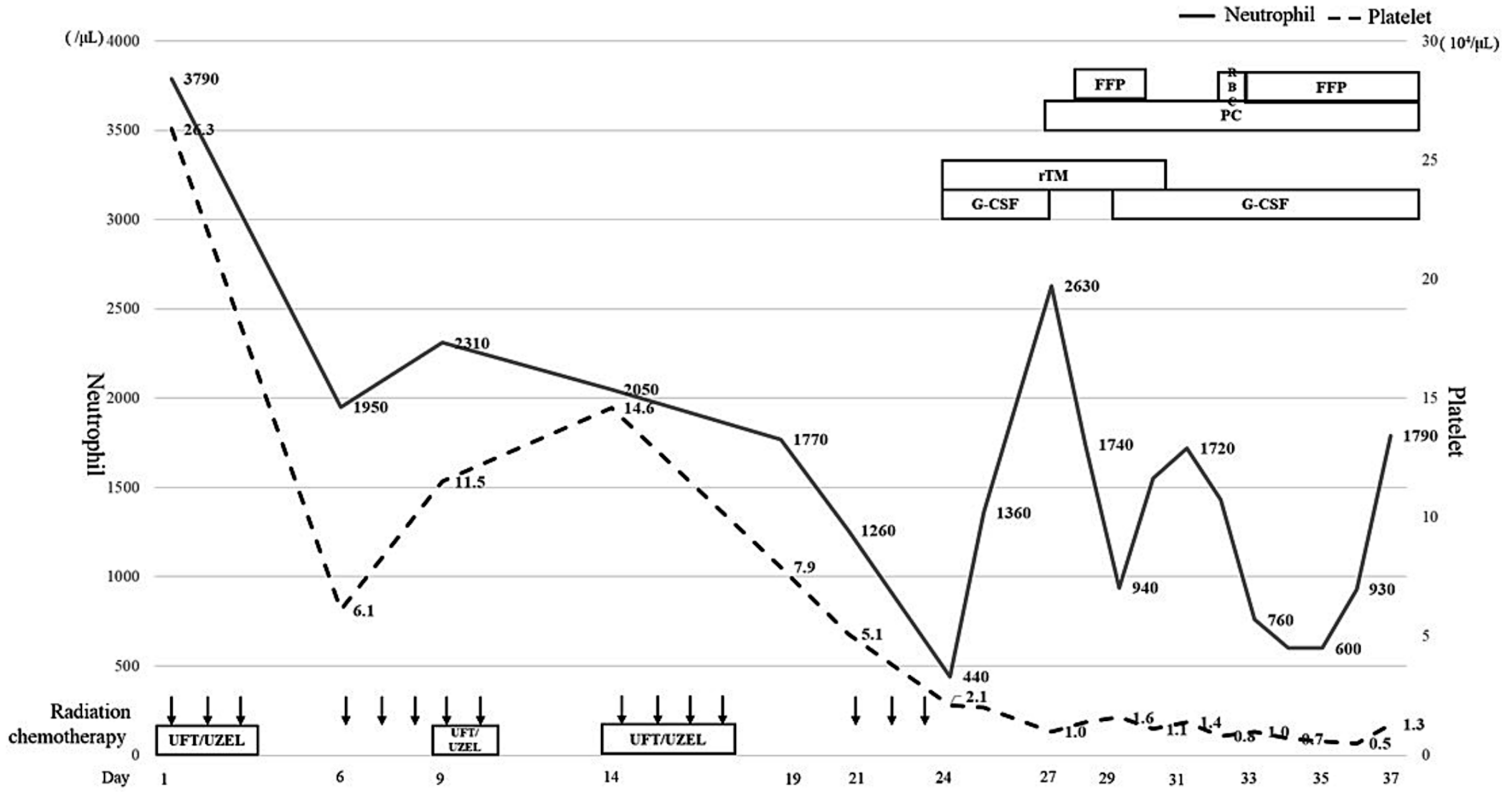
This chart shows the neutrophil (bar line) and platelet (dotted line) counts over the course of treatment. The therapy for febrile neutropenia and transfusion therapy for thrombocytopenia are also presented. RBC, red blood cells; PC, platelet concentrates; FFP, fresh frozen plasma; G-CSF, granulocyte colony-stimulating factor; rTM, recombinant human soluble thrombomodulin.

**Figure 4:**
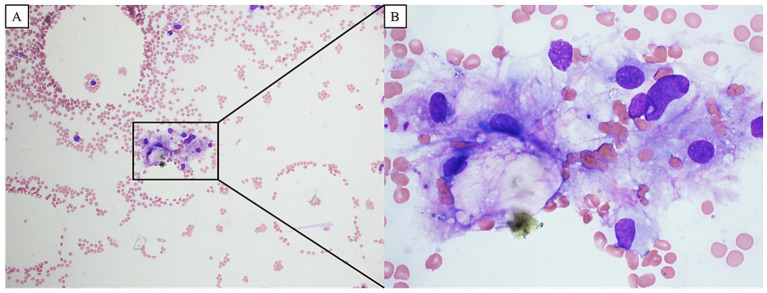
The blood image of bone marrow biopsy showed the hemophagocytosis by macrophages, and the patient was diagnosed with hemophagocytic syndrome as the cause of his poor general condition (**A**: ×100, **B**: ×400).

On the 6th day (Day 6) following the initiation of CRT, anorexia Grade 2 and fatigue Grade 2 were observed based on the Common Terminology Criteria for Adverse Events (CTCAE) v5.0, and tegafur–uracil/leucovorin (UFT/UZEL) therapy was discontinued; this led to an improvement in the severity of anorexia and fatigue, and chemotherapy was restarted on Day 9. On Day 18, the patient developed a fever of 38.3 °C, and blood tests revealed a slight decrease in blood cell counts (white blood cells: 2,330 cells/μL; neutrophils: 1,770 cells/μL). Oral UFT/UZEL therapy was discontinued, oral levofloxacin treatment was initiated on Day 19, and therapy transitioned to intravenous cefepime on Day 21. The fever could not be resolved after that point, and blood tests conducted on Day 24 revealed Grade 4 neutropenia and Grade 4 thrombocytopenia (white blood cell count: 740 cells/μL; neutrophil count: 440 cells/μL; and platelet count: 2.1 × 10^4^/μL) (Figures 2, 3). The patient was diagnosed with febrile neutropenia and disseminated intravascular coagulopathy (DIC) owing to the preoperative treatment he received, and granulocyte colony-stimulating factor (G-CSF) preparation, blood transfusion (red blood cells, platelet concentrates, and fresh frozen plasma), and the antibiotic meropenem were administered, along with recombinant human soluble thrombomodulin (rTM) as DIC therapy. Starting on Day 25, vancomycin was administered in combination with the intravenous meropenem to combat methicillin-resistant *Staphylococcus aureus*. However, the bacteriological testing of the blood and urine failed to detect any bacteria, and the CT imaging did not identify any obvious source of infection, although splenomegaly was identified. The patient’s neutropenia improved with G-CSF administration; however, the platelet transfusions did not lead to an improvement in the degree of thrombocytopenia. The patient continued to exhibit a fever in the 39 °C range; the elevated aspartate transaminase, alanine transaminase, and bilirubin levels led to the suspicion of acute cholangitis, and an endoscopic biliary ductal dilation was conducted on Day 31. However, the patient’s fever, thrombocytopenia, and liver function did not improve, and on Day 34, acute respiratory distress syndrome (ARDS) developed. Non-invasive positive pressure ventilation and prednisolone therapy were initiated, and micafungin, an antifungal agent, was administered for antimicrobial therapy. Neither the blood cultures nor the CT imaging performed at the onset of ARDS were able to identify any infectious agent; therefore, a bone marrow biopsy was performed to investigate the cause of the patient’s fever. Imaging of the bone marrow biopsy specimen revealed hemophagocytosis by macrophages (Figures 4A,B), and the patient was diagnosed with HPS as the root cause of his poor general condition. Blood tests conducted on Day 35 showed elevated levels of lactate dehydrogenase (2,321 U/L), ferritin (58,100 ng/mL, compared to 5,289 ng/mL on Day 25), and triglycerides (469 mg/dL), as well as decreased levels of fibrinogen (57 mg/dL); furthermore, the EBV DNA concentration was 6.22 logIU/mL, and the CMV nucleic acid concentration was 3.2 × 10^3^ IU/mL. On Day 36, treatment with valganciclovir hydrochloride was initiated to combat CMV. Non-occlusive mesenteric ischemia was suspected based on the patient’s bloody stool samples; however, owing to his poor general condition, he was treated conservatively. The patient was intubated for respiratory management; however, takotsubo cardiomyopathy was observed, and his general condition did not improve. He ultimately passed away on Day 37.

## 4 Discussion

HPS is a condition characterized by the phagocytosis of autologous blood cells by macrophages or histiocytes in the bone marrow, spleen, lymph nodes, and other components of the reticular system. HPS can be classified as either hereditary primary HPS or reactive secondary HPS (2). Primary HPS occurs in inherited familial hemophagocytic lymphohistiocytosis disorders and immunodeficiency diseases such as Chediak-Higashi syndrome and X-linked lymphoproliferative disorders (3), whereas secondary HPS may occur in association with various infectious diseases, malignancies, or autoimmune diseases (4). Infectious disease-related HPS has been reported to be caused by viral, fungal, and bacterial (including Rickettsial) infections. Among malignant tumors, malignant lymphomas most frequently lead to HPS. HPS presents with a variety of symptoms, including persistent fever, lymphadenopathy, hepatosplenomegaly, pancytopenia, coagulopathy, liver dysfunction, elevated lactate dehydrogenase levels, hypertriglyceridemia, hyperferritinemia, and elevated soluble interleukin-2 receptor levels (1, 4). The HLH-2004 Diagnostic Guidelines (5) are commonly used to diagnose HPS. In the present case, the patient fulfilled six of the eight criteria, including fever, splenomegaly, thrombocytopenia, hypertriglyceridemia and hypofibrinogenemia, hemophagocytosis based on bone marrow biopsy, and hyperferritinemia.

In this case, the most likely cause of HPS was infection with EBV and CMV. Among infectious agents, EBV is the most common cause of reactive HPS (6). However, other infectious diseases have been reported to be associated with HPS, including CMV, human immunodeficiency virus, human herpes virus, varicella-zoster, parainfluenza, and measles virus. Although malignant lymphoma and other tumors can lead to HPS (4), malignant lymphoma was likely not the cause of HPS in this case, as the patient had already exhibited a complete clinical response to chemotherapy. It is possible that the pathogenesis of HPS in this patient may have been caused by initial lymphocyte exhaustion owing to the chemotherapy administered for the treatment of malignant lymphoma, followed by reactivation induced by EBV and CMV infection owing to the bone marrow suppression induced by the CRT for rectal cancer.

HPS treatment involves steroid pulse therapy and the administration of immunosuppressive drugs to suppress macrophage and T lymphocyte activation while simultaneously controlling the underlying disease. For example, the treatment protocol published in HLH-2004 Diagnostic Guidelines recommends the administration of etoposide, dexamethasone, and cyclosporin A (5). Other studies have reported that continuous hemodiafiltration using a polymethylmethacrylate membrane hemofilter can be efficacious in correcting high cytokinemia in cases in which the levels are difficult to control via medical therapy in combination with background disease treatment (7). In this case, after the diagnosis of HPS, steroid pulse therapy and treatment for CMV infection (valganciclovir hydrochloride) were initiated; however, the treatment was not effective owing to the poor general condition of the patient following the onset of ARDS. The patient presented with fever and thrombocytopenia; although standard treatment was administered for febrile neutropenia, an earlier diagnosis and treatment with HPS in mind could have minimized the likelihood of experiencing a poor outcome.

This patient had a combination of DLBCL and lower rectal cancer. After achieving a clinical complete response to chemotherapy for DLBCL, HPS developed during CRT for rectal cancer, resulting in an unfortunate outcome owing to uncontrolled disease. During CRT for rectal cancer, fever and marked thrombocytopenia were observed; therefore, broad-spectrum antibiotic therapy was initiated, G-CSF was administered, and rTM treatment began, as febrile neutropenia is associated with DIC. However, no obvious source of infection was identified in the cultures or through CT imaging, and little improvement in the patient’s fever and general condition was observed during the course of treatment. Since the HPS diagnosis was based on the results of the bone marrow biopsy conducted after the onset of ARDS, the patient was already in a life-threatening condition by the time HPS treatment was started. Thus, it is important to consider the possibility of HPS caused by viral infection and to ensure diagnosis and treatment are performed in a timely manner when refractory fever of an unknown origin is encountered alongside marked thrombocytopenia during the treatment of a malignant disease. Our study has the limitations of being a single case report, and more detailed investigations into the onset of HPS were not conducted. If we had performed immune system or genetic analysis before starting therapy for this patient, we might have been able to examine the mechanisms underlying the onset of HPS in greater detail.

## 5 Conclusion

In conclusion, this report described a case of life-threatening HPS caused by EBV and CMV infection during preoperative CRT for rectal cancer. When refractory fever of an unknown origin and decreased blood cell counts are observed during the treatment of a malignant disease, HPS should be suspected as a potential cause, and an active examination should be conducted, including bone marrow biopsy, to optimize clinical outcomes.

## Data Availability

The raw data supporting the conclusions of this article will be made available by the authors, without undue reservation.
